# Supplementary material for: Chemical Composition of Essential Oils from Leaves and Fruits of Juniperus foetidissima and Their Attractancy and Toxicity to Two Economically Important Tephritid Fruit Fly Species, Ceratitis capitata and Anastrepha suspensa
Source: Molecules. 2021 Dec 11;26(24):7504. doi: 10.3390/molecules26247504 (PMC8704769; doi:10.3390/molecules26247504)
Supplement: Supplementary file 1 [file molecules-26-07504-s001.zip › molecules-1490032-supplementary.pdf]

molecules

MDPI

(Article)

Chemical Composition of Essential Oils from Leaves and Fruits of *Juniperus foetidissima* and Their Attractancy and Toxicity to Two Economically Important Tephritid Fruit Fly Species, *Ceratitis capitata* and *Anastrepha suspensa*

Mehmet Kurtca [1], Ibrahim Tumen [2,*], Hasan Keskin [3], Nurhayat Tabanca [4], Xiangbing Yang [4], Betul Demirci [5] and Paul E. Kendra [4,*]

1 Department of Chemistry, Faculty of Science, Selcuk University, 42130 Konya, Turkey; mehmet.kurtca@selcuk.edu.tr
2 Faculty of Health Sciences, Bandirma Onyedi Eylul University, 10200 Bandirma, Turkey
3 Department of Forest Products Chemistry, Faculty of Forestry, Bartin University, 74100 Bartin, Turkey; hkeskin@bartin.edu.tr
4 United States Department of Agriculture-Agricultural Research Service (USDA-ARS), Subtropical Horticulture Research Station (SHRS), 13601 Old Cutler Rd., Miami, FL 33158, USA; nurhayat.tabanca@usda.gov (N.T.); xiangbing.yang@usda.gov (X.Y.)
5 Department of Pharmacognosy, Faculty of Pharmacy, Anadolu University, 26470 Eskisehir, Turkey; betuldemirci@gmail.com
* Correspondence: tumen@bandirma.edu.tr (I.T.); paul.kendra@usda.gov (P.E.K.)

Citation: Kurtca, M.; Tumen, I.; Keskin, H.; Tabanca, N.; Yang, X.; Demirci, B.; Kendra, P.E. Chemical Composition of Essential Oils from Leaves and Fruits of *Juniperus foetidissima* and Their Attractancy and Toxicity to Two Economically Important Tephritid Fruit Fly Species, *Ceratitis capitata* and *Anastrepha suspensa*. *Molecules* **2021**, *26*, 7504. https://doi.org/10.3390/molecules26247504

Academic Editor: Luca Valgimigli

Received: 16 November 2021
Accepted: 08 December 2021
Published: 11 December 2021

Publisher's Note: MDPI stays neutral with regard to jurisdictional claims in published maps and institutional affiliations.

Copyright: © 2021 by the authors. Submitted for possible open access publication under the terms and conditions of the Creative Commons Attribution (CC BY) license (https://creativecommons.org/licenses/by/4.0/).

Supplementary material:

Figure S1. Total ion chromatogram of JFLEO
Figure S2. Total ion chromatogram of JFFEO
Figure S3. Juniper (*Juniperus foetidissima* Willd.) tree (Ankara (Beypazari), Turkey)
Figure S4. Fruits and leaves of *J.* foetidissima (Ankara (Beypazari), Turkey)

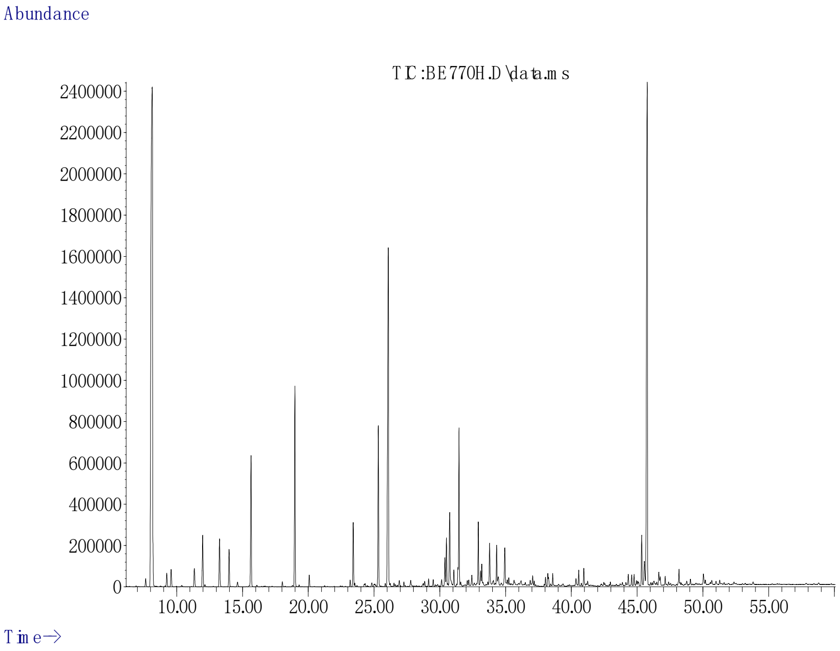

Figure S1. Total ion chromatogram of JFLEO

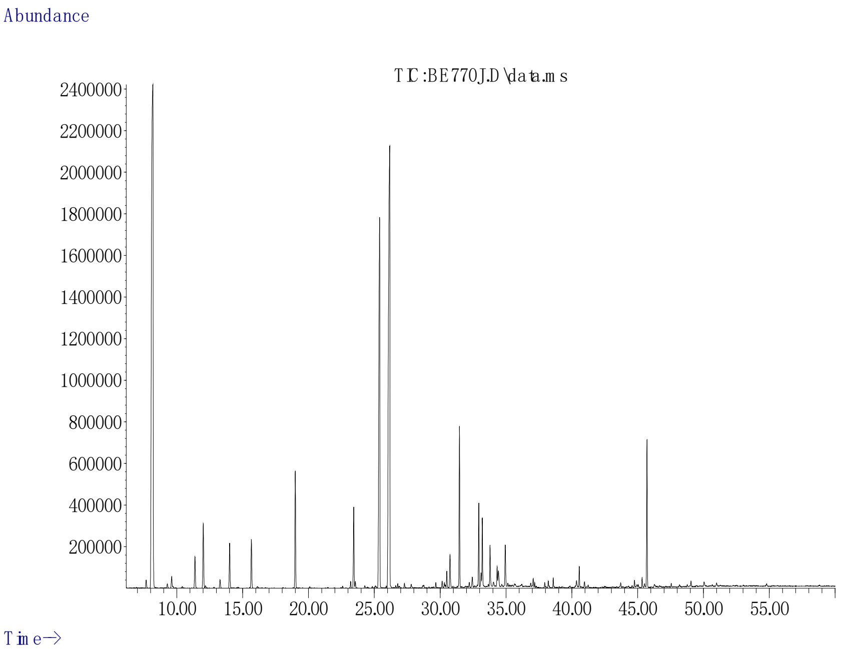

Figure S2. Total ion chromatogram of JFFEO

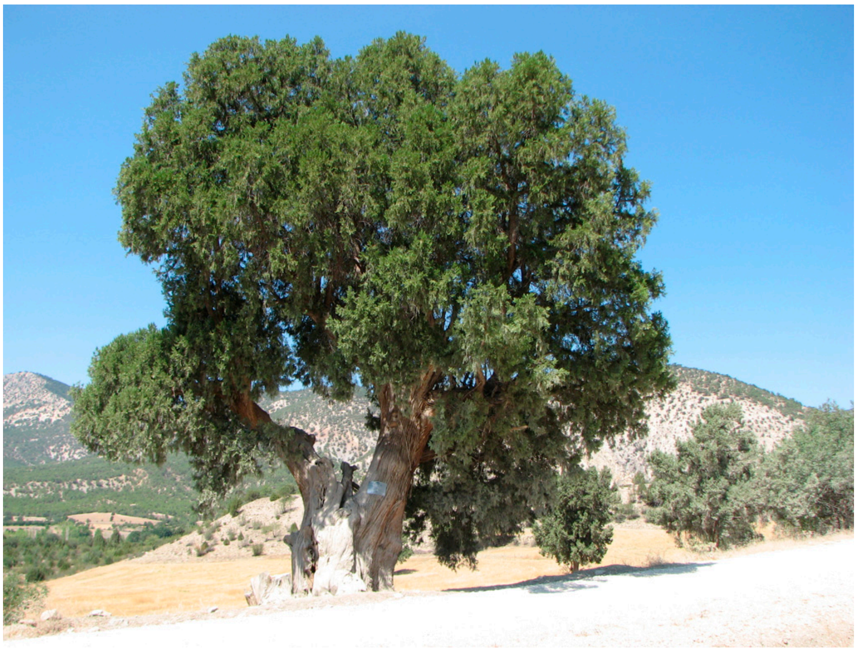

Figure S3. Juniper (*Juniperus foetidissima* Willd.) tree (Ankara (Beypazari), Turkey). Photo credit: I. Tumen (I.T.)

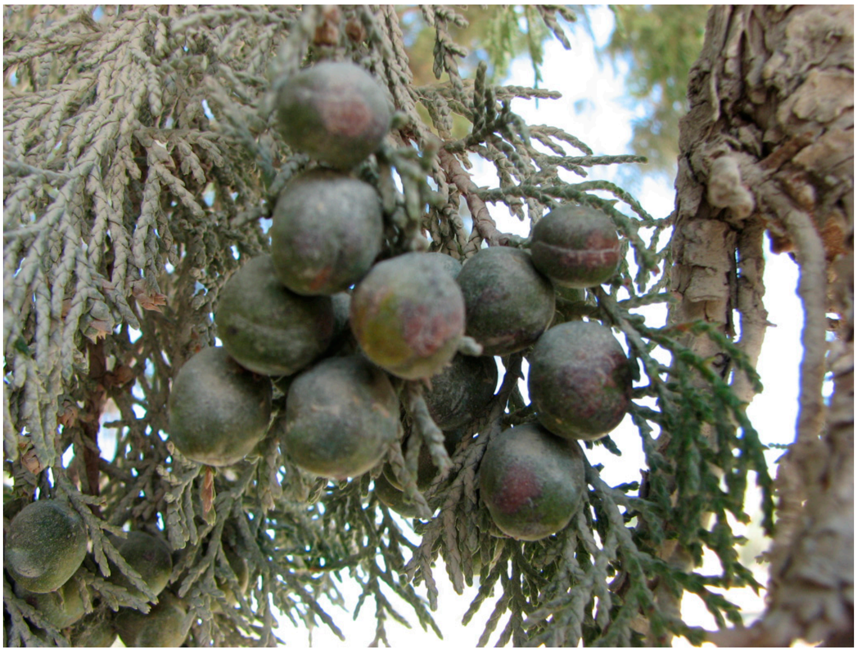

Figure S4. Fruits and leaves of *J.* foetidissima (Ankara (Beypazari), Turkey). Photo credit: I. Tumen (I.T.)
